# How to Implement Digital Services in a Way That They Integrate Into Routine Work: Qualitative Interview Study Among Health and Social Care Professionals

**DOI:** 10.2196/31668

**Published:** 2021-12-01

**Authors:** Janna Nadav, Anu-Marja Kaihlanen, Sari Kujala, Elina Laukka, Pirjo Hilama, Juha Koivisto, Ilmo Keskimäki, Tarja Heponiemi

**Affiliations:** 1 Finnish Institution for Health and Welfare Helsinki Finland; 2 Department of Health and Social Care Systems Tampere Finland; 3 Aalto University Espoo Finland; 4 Oulu University Oulu Finland; 5 South Savo Social and Health Care Authority Mikkeli Finland

**Keywords:** digital services, implementation, health and social care professionals, integration, normalization process theory, interview, social work, health care, focus groups

## Abstract

**Background:**

Although the COVID-19 pandemic has significantly boosted the implementation of digital services worldwide, it has become increasingly important to understand how these solutions are integrated into professionals’ routine work. Professionals who are using the services are key influencers in the success of implementations. To ensure successful implementations, it is important to understand the multiprofessional perspective, especially because implementations are likely to increase even more.

**Objective:**

The aim of this study is to examine health and social care professionals’ experiences of digital service implementations and to identify factors that support successful implementations and should be considered in the future to ensure that the services are integrated into professionals’ routine work.

**Methods:**

A qualitative approach was used, in which 8 focus group interviews were conducted with 30 health and social care professionals from 4 different health centers in Finland. Data were analyzed using qualitative content analysis. The resulting categories were organized under the components of normalization process theory.

**Results:**

Our results suggested 14 practices that should be considered when implementing new digital services into routine work. To get professionals to understand and make sense of the new service, (1) the communication related to the implementation should be comprehensive and continuous and (2) the implementation process should be consistent. (3) A justification for the service being implemented should also be given. The best way to engage the professionals with the service is (4) to give them opportunities to influence and (5) to make sure that they have a positive attitude toward the service. To enact the new service into professionals’ routine work, it is important that (6) the organization take a supportive approach by providing support from several easy and efficient sources. The professionals should also have (7) enough time to become familiar with the service, and they should have (8) enough know-how about the service. The training should be (9) targeted individually according to skills and work tasks, and (10) it should be diverse. The impact of the implementation on the professionals’ work should be evaluated. The service (11) should be easy to use, and (12) usage monitoring should happen. An opportunity (13) to give feedback on the service should also be offered. Moreover, (14) the service should support professionals’ work tasks.

**Conclusions:**

We introduce 14 practices for organizations and service providers on how to ensure sustainable implementation of new digital services and the smooth integration into routine work. It is important to pay more attention to comprehensive and continuing communication. Organizations should conduct a competence assessment before training in order to ensure proper alignment. Follow-ups to the implementation process should be performed to guarantee sustainability of the service. Our findings from a forerunner country of digitalization can be useful for countries that are beginning their service digitalization or further developing their digital services.

## Introduction

### Background

The number of new digital services has been rapidly growing in the health care setting in recent years. Moreover, the COVID-19 pandemic has significantly boosted the implementation of digital services with unprecedented speed and influence [1,2]. The pandemic has also taken the usefulness and potentials of digital services to a whole new level. In doing so, it has also provided an opportunity to add these services to health care systems in the long term [3]. However, despite the pandemic having recently favored the transition to digital solutions [4], digitalization in health care has been slow and complicated, even though major investments have been made [5].

Implementations of digital services tend to fail more often in the health care setting than in other settings because the environment is complex, and therefore the integration into practice is difficult and slow [6-8]. Failure to implement may even lead to a reduction in quality, safety, and efficiency in care [9]. According to the World Health Organization (WHO), guidance for digital health, research, and assessment of the impact of digital service implementations on health care are essential [4]. It is important to identify barriers and success factors when implementing new digital services [10].

A recent systematic review provided a list of barriers and success factors for the implementation of digital services from the organizational point of view [11]. The most mentioned barrier was limited knowledge of the service, and the most mentioned success factor was the services’ ease of use. Resistance from professionals is a major problem for organizations, and therefore it is important to understand their point of view [12]. Health and social care professionals are key influencers in the implementation [13-15] because their attitudes and behaviors influence patients’ capacity to use services and their trust in the services [15,16].

Previous findings show that implementation should include users’ participation at different implementation phases, using champions or other key staff, providing sufficient training and support, and monitoring the use of the system at the early stages of implementation [13,15,17-19]. However, these studies have mainly focused on examining implementation in certain groups of professionals [11,20] or a single digital service in a particular environment [21-25]. There are relatively few recent studies about health and social care professionals' experiences of the implementation of the digital services from a multiprofessional perspective, especially now when the number of implementations has grown and different professional groups have more practical experience about the implementations.

Moreover, now when the COVID-19 pandemic has further accelerated the adoption of the implementations, it is increasingly important to understand how these solutions are integrated into routine work [4]. Digital services are used for varying tasks and purposes, and creating a current overview of the experiences of professionals over a wide range of digital service implementations rather than focusing on 1 specific implementation would be of benefit. In addition, because Finland is the leading country for the third year in a row in digitalization according to the International Digital Economy and Society Index (I-DESI) [26], perceptions from Finnish professionals about the implementations can provide valuable information for many organizations that are further developing their digital services and systems.

### Objectives

The aim of this study is to examine health and social care professionals’ experiences of digital service implementations and to identify factors that support successful implementations and should be considered in the future to secure that the services are integrated into professionals’ routine work. Normalization process theory (NPT) was used as an analytic framework [27]. This enables an understanding of how digital services can be normalized into professionals’ routine work and workflow.

## Methods

### Design and Settings

A qualitative descriptive design with group interviews was used. The design was chosen because it allows information to be collected directly from those who are experiencing the phenomenon under investigation, as in this study from health and social care professionals [28]. We sought to examine health and social care professionals’ experiences with successful implementations by asking them to share hindering and facilitating factors and what should be considered to achieve a successful implementation in routine work. This study followed the Consolidated Criteria for Reporting Qualitative Research (COREQ) guidelines [29].

The interviews were conducted at 4 different health centers located in different parts of Finland. These health centers were selected because they were forerunners in digitalization as they had adopted new ways of working and operating digitally before and during the COVID-19 pandemic. Each of these health centers were either pilots or pioneers in the implementation of various digital services (eg, digital symptom questionnaires, self-management instructions, and remote health care appointments). More examples of the level of each health center’s digitalization and the services it provides can be found in Multimedia Appendix 1.

In Finland, health care services are divided into primary health care and specialized medical care. Municipalities (local governments) operate health centers, which represent citizens’ first points of contact in public health care. Health centers provide a wide scope of primary health care services, such as general practice outpatient care, maternity and child health clinics, health promotion, oral health care, medical rehabilitation, home nursing, and laboratory and basic imaging services, as well as community hospital care. In some health centers, services may also include some specialized care, such as mental health and substance abuse services [30].

### Participants

The participants were health and social care professionals (N=30) working in 4 health centers (Table 1). They were purposely selected by asking clinic managers to recruit volunteers. The inclusion criteria were that the participants had to be health and social care professionals who did client work at health centers that have implemented digital services into their work, and therefore they had recent experience with digital service implementations. Those professionals who had indicated their willingness to participate were contacted via email with information about the study and were asked their willingness to participate in the interviews. All participants provided written informed consent.

**Table 1:** Demographic characteristics of interviewed professionals (N=30).

Category and variables		Value, n (%)
**User group**		
	Registered nurses	8 (27)
	Public health nurses	5 (17)
	Practical nurses	7 (23)
	Physicians	5 (17)
	Social workers	3 (10)
	Social counselor	1 (3)
	Digital counselor	1 (3)
**Gender**		
	Male	3 (10)
	Female	27 (90)
**Age (years)**		
	<30	5 (17)
	30-40	11 (36)
	41-50	10 (33)
	51-60	4 (14)
**Career in this organization (years)^a^**		
	<1	1 (3)
	1-5	15 (50)
	5-10	3 (10)
	10-20	5 (17)
	≥20	3 (10)
**Career in total (years)^a^**		
	<1	1 (3)
	1-5	7 (23)
	5-10	6 (20)
	10-20	6 (20)
	≥20	5 (17)

^a^Not all of the participants answered this question.

### Data Collection

The data were collected with 8 semistructured focus group interviews. The focus group interview method is often used as a qualitative approach when the aim is to obtain data from a purposely selected group of individuals [31], for example, multiprofessional groups such as those in our study. We conducted 2 interviews in each organization. In each focus group, there were 4-6 participants from different professional groups, including physicians, registered nurses, public health nurses, practical nurses, social workers, social counselors, and digital counselors (Table 1). Five of the interviews were conducted face to face, but because the COVID-19 pandemic got worse in Finland, the rest of the interviews were conducted remotely using the Microsoft Teams application. The interviews were performed by 3 interviewers (authors JN, A-MK, and EL) from a research team with previous experience in conducting qualitative interview studies and experiences with digital service implementations.

The questions in the interview guide were based on the literature [11,15,17,18] and defined in collaboration with the research team (Multimedia Appendix 2). The interview guide included questions about the professionals’ experiences with digital service implementations, such as which factors facilitated or hindered the implementations and what kind of suggestions the professionals had for future implementations to ensure that services integrate into routine work. Demographic questions related to age, gender, education, and working years in the current organization and total years of working.

The questions in the interview guide were tested in a pilot interview with 1 health care professional by using the Microsoft Teams application. In addition, participants in the first focus group were asked to rate the understandability and relevance of the questions. No changes were required to the interview guide, so the pilot interview was included in the study with the consent of the interviewees. With the permission of the participants, the interviews were recorded and then transcribed by a transcription company. The transcribed text was generated on 168 pages with a line spacing of 1.15, 11-point font, and the font style Verdana. The duration of the interviews ranged from 41 to 79 min, and the total duration of all the interviews was 501 min.

### Data Analysis

**Figure 1:**
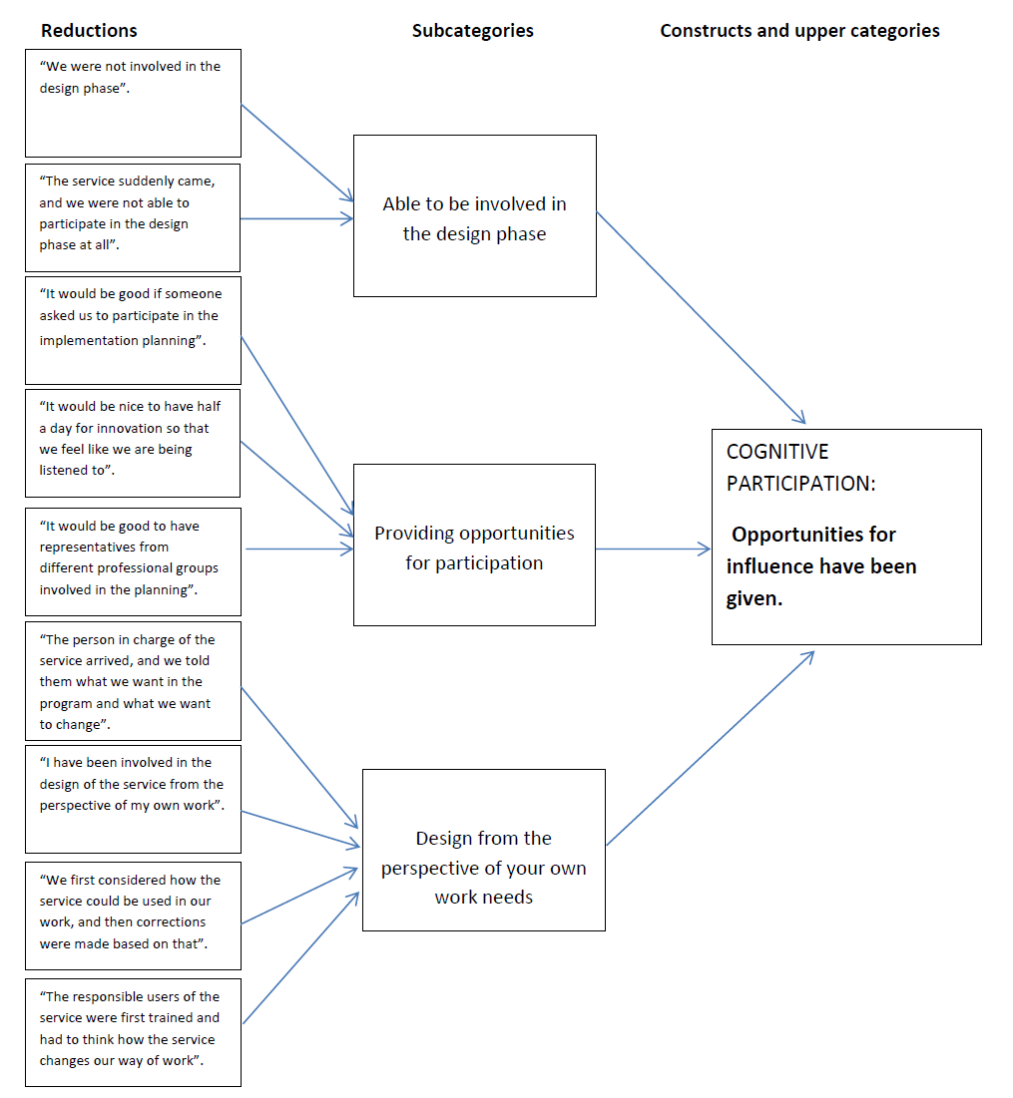
An example of the development of the content analysis process.

We discussed the data saturation after the sixth focus group [32], recognizing that responses began to replicate one another and professionals had similar types of experiences with the implementation. The data were analyzed by using content analysis with an inductive-deductive approach [33]. First, 1 researcher (JN) read all the transcribed interviews a couple of times to form a preliminary image of the data. Then, all the expressions that responded to the aim of the study were extracted from the text and formed into codes (n=224) using ATLAS.ti software (ATLAS.ti Scientific Software Development GmbH). The codes were reductions of professionals’ thoughts. Subcategories were then formed by grouping codes with similar content, which were then formed into upper categories. At this point, 2 other researchers (authors A-MK and SK) looked at the coding and the formed subcategories and upper categories, and discussions were held to reach an agreement on the content and names. The deductive method was then followed, in which the upper categories were divided into 4 different components according to the NPT framework (Figure 1).

We chose the NPT framework because it is a commonly used framework in implementation studies describing how new technologies and other complex interventions are normalized into routine work in health care settings [34]. It is an action theory, which means that it is concerned with explaining what people do rather than their attitudes or beliefs. Thus, it allowed us to understand the key actions that either promote or inhibit the implementation and integration of the services into professionals’ routine work [35]. Moreover, we chose this theory for our study because it can be used to describe and judge the potential of the implementation, but it also has the ability to design and improve complex interventions [27]. We used all 4 of the NPT’s components to obtain an overview of the implementations, contrary to previous studies, which mainly focused on just some of the components [36].

The first component (coherence) seeks to explain how the new service changes work and what are the aims and benefits of the service [27]. The second component (cognitive participation) focuses upon the work undertaken to engage people using the service and get them to buy into it [36]. The third component (collective action) refers to work that enables the implementation to happen [36]. It requires the organization to be supportive and people to have the necessary skills and training to perform the tasks associated with the digital services [27]. The fourth component (reflexive monitoring) includes questions such as whether people try to change the practice to fit their work, how they value the new digital services, and what effects the service has on peoples’ work [27].

### Ethics Statements

The Research Ethics Committee approval (THL/2304/6.02.01/2020) was applied for ethical support from the Finnish Institute for Health and Welfare. The data collected in the study were treated confidentially, and the results are reported in a way that does not identify an individual respondent.

## Results

### Major Findings

We identified 14 practices that, based on the experiences of the professionals, support successful implementation. A detailed description of how the different practices are distributed under the components of the NPT is given in Table 2. The quotation abbreviation meanings are as follows: I=interview and P=participant.

**Table 2:** Good practices when implementing digital services based on NPT^a^ components (n=224).

NPT component and good practices^b^		Mentions^c^, n (%)	
**Coherence (sense-making work): how professionals understand and make sense of the new service (n=38, 17%)**			
	Communication is comprehensive and continuous: The information is multichannel The service presentation reaches everyone. The service has been informed		28 (13)
	The implementation process is consistent: The implementation process needs to be clear There is enough time to get ready for the implementation		7 (3)
	The use of the service is justified: The reasons for using the service are given		3 (1)
**Cognitive participation (relational work): how professionals engage and participate in the service (n=20, 9%)**			
	Opportunities for influence have been given: Professionals are able to be involved in the design phase Professionals are provided opportunities for participation The design is from the perspective of the professionals’ own work needs		15 (7)
	The attitude toward the service is positive: Previous positive experiences toward eHealth implementations occur Professionals show interest in the service Professionals accept the need for the implementation		5 (2)
**Collective action (enacting work): the work that individuals (professionals) and organizations have to do to enact the new service (n=136, 60%)**			
	Support is provided from several fast and efficient sources: Support is given The support model is clear Support is close and easily accessible Support is given by champions Support is received from the work community itself Faster/more efficient sources of support are needed		39 (17)
	Sufficient time is provided for familiarization with the service: Time is provided for familiarization with the service Independent information retrieval and usage learning are required The service must be learned alongside the work A demo version is needed to practice before deployment		36 (16)
	Enough knowledge of the service is provided: Coworkers teach each other with sufficient skills There are no shortcomings in basic technical skills There is a need for nonstop training Sufficient and clear information about the use of the service to support its use is provided Training is systematically planned		31 (14)
	The training is targeted according to work tasks and competence: There is a need for a competence survey Training is targeted according to professionals’ work tasks Training is targeted according to professionals’ skill level/needs		18 (8)
	Various teaching methods are provided: Versatile teaching methods are available Good and clear written instructions are provided Video training is needed to support learning		12 (5)
**Reflexive monitoring (appraisal work): how professionals reflect on or appraise the effects of the services (n=30, 13%)**			
	The service is easy to use: The assumed heavy usability of the program prevents successful deployment Experiences with poor usability affect introducing new programs The service is easy to use The service has no functional weaknesses		12 (5)
	Usage monitoring takes place: There is continuity of deployment monitoring and evaluation		9 (4)
	Giving feedback on the service is possible: The feedback channel is known Sending the feedback forward is smooth		5 (2)
	The service supports work tasks: The service is perceived as useful		4 (2)

^a^NPT: normalization process theory.

^b^The categories inside the NPT’s components are sorted in the order in which the participants mentioned the most.

^c^How many times the participants mentioned this category.

### Coherence: How Do Professionals Understand and Make Sense of the New Service?

The participants considered that the implementation should be prepared by *comprehensive and continuous communication*. They experienced that the communication had failed while announcing that information about the upcoming implementation happened unexpectedly and therefore the presentations had not reached everyone. For future implementations, participants suggested that the communication be done through different communication channels so that it reaches as many employees as possible and avoids uncertainties in the implementation of the service.

Implementation is considered successful when the communication is multichannel, which includes a video, a brochure, and a physical person to talk about the service.

I7, P3

According to the participants, for digital service implementation to be successful, *the implementation process must be consistent*. The implementation process needs to be clear, and the professionals should have enough time to get ready for the upcoming implementation. The participants had previous experiences with how implementations had taken place on a tight schedule, and thereby a lot of ambiguity had been associated with the process. Some of them had previous experiences where they were first told that the service was going to be implemented but later it was canceled and they attended unnecessary trainings. They started to lose their trust in future implementations and changes.

You will lose trust for any change when you experience unclear implementation experiences.

I2, P1

In addition, the participants also felt that it is important *to provide a good justification for using the service*. They felt that telling them why the service was implemented and why it needs to be used gave them the motivation to use it in their routine work. In addition, accepting the digitalization and understanding it as a mandatory way of working were contributing factors. Some of the participants even felt that the time is apt for digitalization and that they must simply go with the flow. They also considered it important that the benefits of using the digital service (from the perspective of their own work) were emphasized.

Implementation is enabled by the fact that the employee [themselves] perceive the service as useful and good in [their] own work.

I3, P1

### Cognitive Participation: How Do Professionals Engage With and Participate in the Service?

The participants noted that in good implementation, champions who have an interest and additional training in the service should be involved in implementation from the beginning. They also highlighted that it is important to give everyone, not only the champions, *an opportunity to influence and participate* by giving insight for the service of one’s own work needs. The participants suggested that implementation be facilitated by involving professionals from different professional groups in the design phase so that everyone would have a voice. In addition, the participants mentioned that it is important that they have been given the option to use working hours when participating in the design phase of digital services.

Usually, we have no time to innovate. It would be nice if there was, for example, half a day for development, which would give the staff a voice.

I2, P3

Ensuring that the professionals have a *positive attitude toward the service* enables successful implementation according to the participants. If the professionals had prejudices or negative attitudes toward the digital services, they were reluctant to use them. The participants expressed that usually these negative emotions were consequences of a lack of involvement in the design phase. Prejudices were also often related to the usability of the service. It was feared to be too difficult to use or the professionals simply had bad experiences with previous implementations.

Prejudices can negatively affect successful implementation because you may have some perception or fear that the service [that] is being implemented is too difficult to use.

I7, P3

### Collective Action: The Work That Professionals and Organizations Must Do to Enact the New Service

The participants pointed out that *support should be available from a variety of sources*. Lack of support or the support model being unclear was found to hinder successful implementation. They noted that everyone should know how the support is provided, whom to contact in the case of a problem, and where to find all the related contact information. The participants had 2 views on the source of support. Some believed that support required a person physically present who could be approached quickly in problem situations. Especially after the first weeks of implementation, this was considered important. For some, remote support was thought to be adequate, as this would allow support to remotely connect the user’s computer, if necessary, thus quickly supporting the user in the event of a problem. Nevertheless, what was considered to be the most important thing was that the support should be close and readily available.

The person providing the support does not have to be physically present, but it would be good to have support remotely that is easily and quickly available so that [they] can remotely show how to do these things with the service.

I8, P3

In addition to the support provided by the organization, support was often received from one's own workplace, most often from one's own colleagues. Some of the participants had experiences of champions supporting the use of the service. In addition to receiving support from their own colleagues, some of the participants also received support from their own supervisors. However, only a few considered it to be important that the supervisors support employees in the implementation process. For them, the attitude of supervisors toward digital services was the more substantial contributing practice to successful implementation.

Most of the participants mentioned that there should be *enough time to get familiar with the service.* They experienced that they had not been provided enough time to get to know the service but had to learn to use it alongside their work while the patient was at the reception. They suggested that by having a demo version, they would get an opportunity to practice the service independently or with colleagues before using it with patients. It would also give them the chance to practice it in peace and when it suits them best. However, some of the participants saw that the use of the service would be learned over time while working or through mistakes at the latest.

When you fail enough times, you will get it right eventually. You will learn from your mistakes.

I6, P3

According to the participants, well-planned and scheduled training regarding the use of the service, as well as equal opportunities and time to participate in these training sessions, are important. The training should also be nonstop so that it would train as many employees as possible and even the shift workers could attend. The participants also pointed out that having *enough knowledge on how to use the service* is important for the success of the implementation. They believed that having enough knowledge would help them achieve confidence in using the service. The participants described problematic situations where the coworkers had taught other coworkers with insufficient knowledge. Furthermore, with such experiences, they mentioned that effective and clear information about the use of the service ensures that it is used correctly.

In addition, 1 of the key practices that the participants pointed out was that the service provider or the organization should *provide different training methods* for employees. One-sided training methods prevented successful implementation. For example, watching training videos alone did not guarantee sufficient skills to successfully use services. However, some of the participants experienced that video training enabled recounting whenever they needed it. In addition to various training methods, participants mentioned that it is important to have, especially after its implementation, written instructions on how to use the service.

The participants hoped that their skills could be assessed to map their training needs. Targeting training according to the level of competence would be useful, as some people may need to learn more basic technical skills, while others already master them well and may be able to cope with shorter training sessions. It was also mentioned that the individuals who embrace the program more easily, such as recent graduates, may find the video training enough and no other forms of training will be needed. The importance of targeting training according to the competence requirements set by the job tasks was also emphasized.

I would have wished for targeted training for my own professional group because now they have been general. It would be more efficient if the [training sessions] were more targeted, so you could focus on the things you need in your own work.

I8, P2

### Reflexive Monitoring: How Do Professionals Reflect on or Appraise the Services’ Effects?

The participants noted that after the implementation, it is important that the service be perceived to work well, because experiences of poor usability were believed to jeopardize successful implementation. Thus, the *service should be easy to use* and should not have usability vulnerabilities.

Usability and especially the ease of using the service plays a huge role if you want the implementation to be successful.

I2, P1

In addition, after the implementation of the service, *monitoring its use* was also considered important. This meant, for example, regular monitoring of the correct use of the system and the use of all included features. The participants were concerned about the misuse of the system due to a lack of sufficient training and monitoring of everyday use.

The problem is that no one comes back and asks if you have learned to use the service; a follow-up visit is needed.

I7, P3

The participants also pointed out the importance of getting an *opportunity to provide feedback* on the new service after the implementation and that the feedback be used to develop the service. It was essential for the functioning of the feedback channel that it be known by everyone and that the feedback process be perceived as smooth. If it was not perceived as being smooth, there was no desire to give feedback.

If you come up with a good idea, taking it forward was not made easy; if it takes a lot of time, it is often left undone.

I2, P2

According to the participants, the use of the digital services that are being implemented should be *useful for one's own work*. The service was not used nor recommended, for example, to patients if the participant considered the service unbeneficial for one's own work.

## Discussion

### Principal Results

This qualitative study identified factors that support successful digital service implementations and should be considered in the future to make sure that the services are integrated into health and social care professionals’ routine work. According to professionals’ implementation experiences and by using the NPT framework, we suggest 14 practices that should be considered when implementing new services into professionals’ clinical work.

To get professionals to understand and make sense of the new service, (1) the communication related to the implementation should be comprehensive and continuous and (2) the implementation process should be consistent. (3) A justification for why the service is going to be implemented should also be given. The best way to engage professionals with the service is (4) to give them opportunities to influence and (5) to make sure that they have a positive attitude toward the upcoming service. To enact the new service into professionals’ routine work, it is important that (6) the organization take a supportive approach by providing support from several easy and efficient sources. The professionals should also have (7) enough time to become familiar with the service and have (8) enough know-how about the service. The training should be (9) targeted individually according to skills and work tasks, and (10) it should be diverse. The impact of the implementation on the professionals’ work should be evaluated. The service (11) should be easy to use, and (12) usage monitoring should happen. An opportunity (13) to give feedback on the service should also be offered. Moreover, (14) the service should support professionals’ work tasks.

### Comparison With Prior Work

According to our study, to ensure that the service makes sense (the NPT’s component coherence) for the users, the communication related to the implementation should be comprehensive and continuous. In earlier studies, the importance of good information has been highlighted when implementing digital services [14,17,18,37]. However, previous studies do not mention specifically what kind of good communication would best support professionals in their work. In our study, the professionals suggested that the communication should happen from a variety of different channels so that it reaches everyone, including shift workers. Our results highlight that when the implementation process happens under a tight schedule, such as during the COVID-19 pandemic, the importance of comprehensive and continuous communication increases.

In our study, the professionals also highlighted that it is important to justify why the service is going to be implemented in order to ensure that it makes sense for the users. The benefits of using the service seemed to be especially important from the perspective of the professionals’ own work. Sanders et al [38] presented in their study that usually the difficulty in implementations is a failure to clarify coherence to the users. If the professionals fail to understand the way of working as helpful and relevant, they may be unwilling to use it. In addition, May et al [37] highlight the importance of coherence; if there is a desire for the service to be normalized into the professionals’ work, it is important that the service make sense for the users [27,36]. In the review by Mair et al [19], the sense making was not highlighted as being that important when implementing eHealth systems. However, it is good to note that this review was conducted 10 years ago.

Some earlier studies have pointed out that involvement is 1 of the key contributing factors when implementing new digital services [17-19]. In addition, the NPT [27,36] highlights the importance of engagement and participation (cognitive participation component) with the service to get it normalized into routine work. In our results, the professionals underlined the importance of everyone getting an opportunity to influence and participate in designing the service. Our results therefore support previous results; however, the main problem according to our results is that time for involvement is not given to professionals. The heavy workload and the lack of staffing of health and social care settings have increased in recent years, which may influence the time given for innovation and design [39].

According to our results, to make sure the enactment of the work happens (collective action component), the support should be provided from a variety of fast sources. However, it was interesting that the professionals did not highlight the importance of supervisors’ support, whereas previous studies have recognized the lack of support from supervisors as 1 of the major barriers to implementation [17,18,40]. However, we found that the positive attitude toward the service is more important. In addition, enactment of the work happens if people have the necessary skills and training to perform the tasks associated with the services. In our study, it was important for the professionals to have enough knowledge about the service and time for its familiarization. In previous studies, the lack of time [11,18] and the lack of information [18,19] were also experienced as inhibiting factors.

In our study, it was important to use various teaching methods and training, which was targeted according to work tasks. Professionals also suggested to target the training according to the competence assessment. Previously, the training and competence of professionals have been found to be key factors when implementing new digital services [11,18,19], but competence assessments have received less attention. Mair et al [19] mentioned the importance of training, but they did not specify what kind of training would be required. Therefore, more information is needed in the future about what, how, and how much training should be provided.

In our study, it seems that the evaluation of a new digital service depends on how well the service supports professionals’ work tasks and whether users perceive the service use usable (reflexive monitoring). Our results suggest that follow-ups are important, because after the implementation, the users expected someone to monitor whether they were using the services in an appropriate manner. Gagnon et al [17] also found that monitoring the use of the system should happen at the early stages of implementation to ensure immediate response to users’ feedback [17]. It is important that the organizations and service providers keep on engaging with the service users after the implementation has taken place. This also provides an opportunity to ensure that the service is used correctly. In addition, in our study, being able to give feedback about the service was considered important because it gave the users the feeling that they can influence. This also gives the organizations and service providers an opportunity to further develop the service based on the users’ needs.

The NPT framework seemed to be a suitable choice for our research because our findings were well interpreted with the components of this framework. The NPT offered us a useful tool for organizing the important practices involved in the data, which enabled the development of recommendations for future implementations. We had difficulties sometimes in understanding and applying coding to some of the NPT components, especially coherence and cognitive participation, which are more related to time before implementation. However, even when we used all 4 components, collective action frequently got many mentions, maybe because it describes the enactment of the work most comprehensively. May et al [37] suggested that for future studies, it is important to connect collective action much more closely to the context in implementation studies.

### Strengths and Limitations

The strengths of this study include multilevel information about experiences from different professional groups when implementing a different kind of new digital service in the era of the COVID-19 pandemic. The fact that a fairly large number of interviewees (n=30) took part can also be considered a strength. Our results are from the forerunner country in digitalization and from 4 different health centers that are pioneers in digitalization and located in different parts of Finland. Thus, we were able to obtain important information from pioneer organizations for many organizations that are further developing their digital services and systems. A qualitative research method was able to give us a more in-depth overall picture of the situation during the era of COVID-19.

Credibility was established in this qualitative study by following the criteria of reliability presented by Lincoln and Cuba [41]. To establish confidence that the results were true, we collected data generated by 3 interviewers, 1 of whom was a more experienced interviewer. In the Results section, we used quotations to make sure the results were authentic. Credibility and interrater reliability were increased by having several sessions of peer debriefing with the research team about the interviews, the analyses process, and the name of the codes. Dependability was guaranteed by describing the study methods, data collection process, and analysis process as thoroughly as possible. Confirmability was guaranteed by collecting data from different groups of professionals, who were performing different kinds of tasks in a health and social care setting. Transferability was guaranteed by purposely sampling different kinds of professionals to participate in the focus group interviews.

This study had some limitations. One limitation was that because the COVID-19 epidemic got worse during data gathering, we had to conduct some of the interviews using the Microsoft Teams application instead of face-to-face interviews. The focus group interviews’ aim is to get people to talk in a group, and with Microsoft Teams, it was more difficult and required more effort from the interviewers. However, fortunately, our interviewers were experienced and were able to plan a strategy for promoting discussion. Interrater reliability was also 1 limitation, because if we had performed the coding by double-coding it, we could have compared the unity of the coders more closely instead of only discussing it [42]. One more of the limitations was that the interviews were conducted in Finnish health centers; compared to other countries, Finland is ahead in digitalization and it can therefore influence implementation attitudes and experiences. So, transferability to other countries must be done with caution, especially related to countries with a low level of digitalization. The interviews were conducted in health centers, where there can be certain types of digital services in use, and therefore the results may not be transferable to other health care contexts. However, a previous review (eg, Mair et al [19]) showed corresponding results even when the environment varied. In addition, given that these 4 health centers were purposely selected because they were advanced in digitalization, it may have also influenced the results. Finally, 1 of the limitations was that most of the professionals who attended the interviews were from the health care sector, and therefore future studies about social care professionals’ experiences with digital service implementations should be conducted.

### Conclusion

In this study, we examined health and social care professionals’ experiences with digital service implementations and identified factors that support successful implementations and should be considered in the future to ensure that the services are integrated into professionals’ routine work. Based on the results, we suggested 14 practices for organizations to consider when implementing new digital services. Due to practical reasons, such as limited time and resources and a high number of implementations in organizations, it may not be realistic to expect all the practices to be fully executed. However, these practices can guide organizations to find appropriate ways to support professionals and help organizations to pursue successful implementations. Our findings can be useful for countries that are beginning their service digitalization or further developing their digital services. For future studies, it is essential to examine implementations in a different phase of the process. The digital services add workload on already busy schedules [17], and thus, it would be beneficial to study how the implementations influence professionals’ well-being at work.

## Appendix

Multimedia Appendix 1 Description of the level of digitalization in studied health centers.

Multimedia Appendix 2 Interview guide.

## Abbreviations

COREQ: Consolidated Criteria for Reporting Qualitative Research
I-DESI: International Digital Economy and Society Index
NPT: normalization process theory
WHO: World Health Organization
