# Supplementary material for: How to Implement Digital Services in a Way That They Integrate Into Routine Work: Qualitative Interview Study Among Health and Social Care Professionals
Source: J Med Internet Res. 2021 Dec 1;23(12):e31668. doi: 10.2196/31668 (PMC8686404; doi:10.2196/31668)
Supplement: Multimedia Appendix 1 [file jmir_v23i12e31668_app1.docx]

Multimedia Appendix 1. Description of the level of digitalization in studied health centers.

| **Health center** | **Examples of digital services provided** |
| --- | --- |
| Health center 1 | Electronic health/medical records, national Kanta services (including access to patient and medical records), patient information systems, laboratory and imaging systems, decision support systems, remote receptions, digital symptom checkers, electronic messaging, online appointment booking |
| Health center 2 | Electronic health/medical records, national Kanta services (including access to patient and medical records, acting behalf of someone else), online appointment booking, access to personal medical records, patient-reported medical history, electronic messaging, digital symptom checkers, self-management instructions, remote healthcare appointments |
| Health center 3 | Electronic health/medical records, national Kanta services (including access to patient and medical records), laboratory and imaging systems, decision support systems, remote receptions, digital symptom checkers, electronic messaging, online appointment booking |
| Health center 4 | Electronic health/medical records, national Kanta services (including access to patient and medical records), OmaMehiläinen service (for example 24/7 remote receptions, online appointment booking, electronic messaging, digital coaching), digital symptom checkers, Forsante service (automates routine anticoagulation therapy), decision support systems |
