# Supplementary material for: How to Implement Digital Services in a Way That They Integrate Into Routine Work: Qualitative Interview Study Among Health and Social Care Professionals
Source: J Med Internet Res. 2021 Dec 1;23(12):e31668. doi: 10.2196/31668 (PMC8686404; doi:10.2196/31668)
Supplement: Multimedia Appendix 2 [file jmir_v23i12e31668_app2.docx]

Multimedia appendix 2. Interview guide

**Background questions:**

- Age
- Gender
- Education
- Work experience in current job
- Total work experience

**Interview questions:** Professionals' experiences of implementing the eHealth service:

- How has the implementation of the eHealth service proceeded in your workplace? (How did it begin and how did it proceed, describe the process, what is the current situation?)
  - How did the change in the work process take place in practice?
- What do you think has contributed to the implementation of the eHealth service and what has hindered it?
  - When was the implementation successful?
  - When did the implementation fail?
  - What factors have you found useful when implementing the service?
  - What factors do you find challenging when implementing the service?
- What kind of support have you received for the implementation (from your supervisor / in your workplace / in general)?
  - What kind of support / orientation would you like to receive?
    - Have you personally experienced that you have received adequate information / training about the service?
    - Have you been supported / encouraged in your use of the service?
    - Have you received information on where to get help with service when problems occur?
- Are there any good practices you could recommend for future implementations?
